# Nocturnal visual hallucinations in patients with disorders of arousal: a novel behavioral and EEG pattern

**DOI:** 10.3325/cmj.2022.63.438

**Published:** 2022-10

**Authors:** Valentina Gnoni, Iain Duncan, Danielle Wasserman, Sean Higgins, Panagis Drakatos, Adam Birdseye, Laura Pérez-Carbonell, Alexander Nesbitt, Michalis Koutroumanidis, Guy Leschziner, Ivana Rosenzweig

**Affiliations:** 1 Sleep and Brain Plasticity Centre, Department of Neuroimaging, Institute of Psychiatry, Psychology & Neuroscience, King's College London, London, UK; 2 Sleep Disorders Centre, Guy's Hospital, London, UK; 3 Department of Neurology, Guy’s and St Thomas’ NHS Foundation Trust, London, UK; 4 Faculty of Life Sciences and Medicine King’s College London, London, UK; The first two authors contributed equally

## Abstract

**Aim:**

To investigate clinical and video-polysomnography (VPSG) findings of hallucinatory experiences in patients suffering from disorders of arousal (DOA) in the absence of other pathologies.

**Methods:**

The authors retrospectively reviewed the records of 370 adults with DOA. Thirty (8.1%) patients concomitantly reported complex nocturnal visual hallucinations. VPSG recordings were scrutinized, and motor behavioral and electroencephalogram (EEG) patterns were classified according to previous descriptions of DOA.

**Results:**

Thirty DOA patients also reported seeing images of objects, people, and animals; either distorted, static, or mobile. The images disappeared with increased illumination in 80% of patients, and 23.3% reported preceding dream imagery. In addition to the classical DOA patterns on VPSG, a distinct pattern of behavioral and EEG manifestation associated with complex hallucinatory episodes was identified in 16 (53.3%) DOA patients. This consisted of low-voltage mixed-frequency EEG activity before eye opening that persisted while patients were observed staring or visually tracking before the onset of motor behavior.

**Conclusion:**

A novel, distinct behavioral and EEG pattern in patients with DOA and history of reported complex nocturnal visual hallucinations was identified. This may represent a unique phenotype of dissociation between sleep states that merits further investigation.

Disorders of arousals (DOA) are a group of non-rapid eye movement (NREM) parasomnias believed to arise from an incomplete arousal from slow-wave sleep (SWS). Inappropriate or reduced responsiveness to external stimuli and partial to complete amnesia are commonly reported (1). These are believed to reflect an underlying dissociative state where sleep and wake coexist across different brain regions (2-7). Frequently, individuals suffer from more than one DOA subtype, and parasomnias emerging from different sleep stages can occur in the same individual (8). Classically, DOA were believed to be characterized by limited or absent dreams and cognition, but several recent studies have cast doubt upon this view, reporting several cases of mentation but also complex narrative, structured dreams during the episodes of DOA (9-11). Some authors have additionally proposed that the brief scenes of mentation during DOA could be more similar to hallucinations from slow-wave sleep than to typical dreams (12).

Historically, there is a lack of clear definition and distinction between dreams and hallucinations (13-15). This may be due to their similarities, a possible similar pathophysiological mechanism, and to the difficulty in being described by patients and how these descriptions are interpreted (15).

Specifically, hallucinations are defined as sensory perceptions that occur in the absence of an actual external stimulus (14). Classically, sleep-related hallucinations are associated with sleep onset or sleep offset (hypnagogic and hypnopompic) (1), and often within the boundaries of REM-related phenomena that may be normal (16) or may be related to central disorders of hypersomnolence (17). In addition, a subtype of sleep-related hallucinations have been defined as complex nocturnal visual hallucinations (CNVH), which are phenomena that occur following an abrupt arousal and are associated with complex vivid images and are also known to occur in the presence of other neurological/medical disorders (1).

Besides this clear definition of sleep-related hallucinations, some hallucinatory experiences have been described in association with other sleep disorders. In fact, comparative experiences have been effectively reported in the literature in conjunction with sleepwalking and other complex behaviors (18-21).

Visual hallucinatory experiences occurring during DOA episodes have been sparsely described in the literature. Therefore, the aim of this study was to investigate the clinical and video-polysomnographic features of hallucinatory experiences in a cohort of patients with DOA in the absence of other pathologies, including neurological, psychiatric, and eye disorders.

## Methods

We retrospectively reviewed patients’ records in the sleep disorder center’s clinical database between August 2017 and May 2020. Among 370 adult patients with a diagnosis of DOA, 30 patients (mean age 33 ± 6.2 years, 21 women) were identified who concomitantly presented with a complaint of nocturnal visual hallucinations. Patients were excluded if they reported hypnagogic and hypnopompic hallucinations occurring in isolation or associated with sleep paralysis or other features of REM dysregulation and central disorders of hypersomnolence. Patients with psychiatric, neurologic (eg, neurodegenerative disorders and other disorders that can present with hallucinations), and eye diseases were also excluded.

### Polysomnography evaluation

All patients underwent a single-night videopolysomnography (VPSG) recording in the sleep center. This included six electroencephalogram (EEG) channels (F3/A2, C3/A2, O1/A2, F4/A1, C4/A1, O2/A1), bilateral electrooculogram (EOG), submental and bilateral anterior tibialis electromyogram (EMG) (20 patients). Ten patients underwent extended 10-20 montage EEG recording (EEG; FP1, FP2, F7, F3, Fz, F4, F8, T3, C5, Cz, C4, T4, T5, P3, Pz, P4, T6, O1, O2) due to the clinical judgment of their respective physician. Respiration was monitored by using nasal cannula with nasal pressure transducer, thoraco-abdominal respiratory inductance plethysmography, and pulse oximeter. Studies were scored by expert somnologist technologists according to AASM criteria (22).

### Clinical review

The cases were further comprehensively reviewed through analysis of medical records and VPSG recordings. All recordings were additionally reviewed in the multidisciplinary team meeting with neurophysiologists in order to rule out any underlying epileptic abnormalities. We reviewed morning questionnaires given to all patients following their sleep study, as per standard sleep center’s protocol. Morning questionnaires are semi structured, self administered, and contain open-ended questions with elements of the Hall and Van der Castle dream coding system (23). Items relating to recall of events and specific questions relating to associated imagery, sensory perception, and emotions at the time of events are included (Supplementary Table 1(Supplementary material 1)).

Patients’ informed consent was obtained. The study was approved by the Guy’s and St Thomas’ NHS Trust institutional review board on human research (Project No 11378, GSTT NHS, UK).

### Statistical analysis

Clinical and VPSG data were compared with the Mann-Whitney U-test. Categorical variables were compared with the χ^2^ test. Statistical significance level was set at *P* < 0.05. The analysis was performed with IBM SPSS Statistics, version 23 (IBM Corp., Armonk, New York, USA).

## Results

### Clinical/behavioral manifestations

All 30 patients presented with a history of DOA. In particular, 22 (73.3%) reported sleepwalking, 22 (73.3%) reported sleep terrors, 3 (10%) reported confusional arousals, and 19 (63.3%) presented also with sleeptalking. One patient was taking a β-adrenergic receptor-blocking medication and one was taking selective serotonin reuptake inhibitors. Nine patients (30%) had a history of anxiety, and three patients (10%) had a history of depression. In addition, the following comorbid sleep complaints were also reported: insomnia (five patients), bruxism (one), excessive daytime sleepiness (three), suspicion of obstructive sleep apnea (OSA) (two), restless legs syndrome (three), sleep paralysis (three), and nightmares (four). All relevant clinical and demographic findings are shown in Table 1.

**Table 1:** Demographics and clinical presentation (N = 30)*

Demographics	N	%
Age, years, mean (SD)	33	6.2
Body mass index, kg/m^2^, mean (SD)	24	4
Epworth sleepiness score, /24, mean (SD)	8.5	6.7
Male	9	30
Female	21	70
**Event frequency**		
high (>1 per night)	5	16.7
moderate (<1 per night, >3 per week)	7	23.3
low (>1 per week, <3 per week)	15	50
very low (<1 per week)	3	10
**Parasomnias**		
past reports of DOA hallucinations	30	100
sleepwalking	22	73.3
sleep terrors	22	73.3
confusional arousals	3	10
sleeptalking	19	63.3
childhood history	23	76.7
**Other presenting sleep complaints**		
none	16	53.3
history of insomnia	5	16.7
history of nightmares	4	13.3
history of sleep paralysis	3	10
history of RLS	3	10
history of suspected OSA	2	6.7
excessive daytime sleepiness	3	10
history of bruxism	1	3.3
**Hallucination characteristics**		
people/shadows of people	27	56.7
insects	10	33.3
animals	5	16.7
objects	4	13.3
disappear with illumination	24	80
interaction with images	16	53.3

*Abbreviations: OSA – obstructive sleep apnea; RLS – restless legs syndrome; SD – standard deviation.

All 30 patients had a history of experiencing episodes of nocturnal visual hallucinations. Typically, these manifested as seeing people or shadows of people (56.7%), objects (13.3%), animals (16.7%), or insects, in particular spiders (33.3%), both stationary and mobile. Images were commonly reported to disappear with increased illumination (80%), and 53.3% of the patients described interacting with the images. Episodes were reported to last generally a few minutes; the frequency of the events ranged between at least once per night to once every two months. Notably, all hallucinatory episodes were reported as visual and monomodal; the images were mute, and no other modality of sensations, such as tactile, olfactory, or auditory, was reported.

Some disturbing imagery was described, including shadows or people with distorted or featureless faces standing immobile in the corner of the room or moving toward the patients. Human characters could be in the form of children, men, or women, usually unknown to the patient, and they could be standing over the patient or sitting on the edge of the bed. These figures were sometimes also reported to interact with the environment. For example, patients reported that they had seen people rummaging through their belongings. Other examples included seeing animals, cats, mice, or insects crawling on the walls, ceiling, or even the bed. Another patient reported that a large spider, the size of a cat, was sitting on their chest. Objects hanging from the ceiling or emerging from wardrobes, mirrors, or cupboards were also described. Macropsia was noted; for example, one patient reported a spherical microphone lowering down from the ceiling and increasing in diameter as it was getting closer. Seven (23.3%) patients were able to recall dream imagery preceding the hallucination episodes, while the remainder were unsure or had no recall of previous dreams (76.6%). Interestingly, dream imagery was commonly related to the subsequent visual hallucinatory images but no underlying unfolding plot or narrative story was recalled.

Patients reported only a partial insight, with hallucinatory images initially generating significant fear and distress. This would subside after a few seconds or minutes, when they became aware that they were experiencing their “visions”. This usually coincided with presumed full wakefulness and awareness, and the patients would turn on the light and, on occasion, leave the bed to investigate.

### Videopolysomnography findings

**Figure 1:**
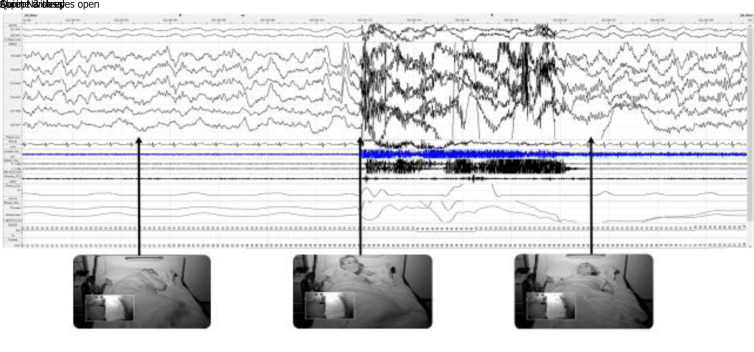
Typical disorder of arousal episode. Upper figure depicts a 30-second epoch from video-polysomnography (VPSG) showing the episode arising from N3 sleep. Motor activity onset corresponds with the electroencephalogram (EEG) artifact, increased electromyogram (EMG) tone on submental and anterior tibialis EMG and movement artifact on respiratory effort bands. Following the resolution of the artifact there is clear intermixed alpha/delta activity in the frontal and central regions, with the eyes remaining open. Lower sequence presents snapshots of the event. The patient is in N3 sleep then suddenly raises her head and trunk with eyes open and looks around the room before lying back down in the supine position with eyes open.

**Figure 2:**
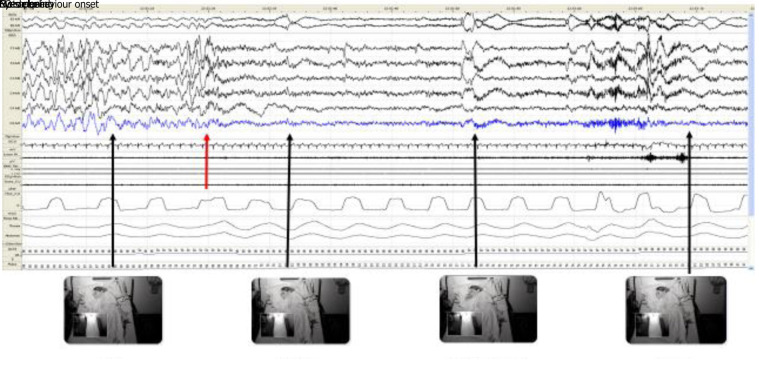
Hallucination episode arising from N3 sleep. Upper figure represents a 60-second epoch of video-polysomnography (VPSG) recording to demonstrate the duration of the event. Following arousal (red arrow) there is a change in electroencephalogram (EEG) to low voltage, mixed frequency, then a 6-second delay to eye opening. A further 12 seconds elapse before motor behavior onset coinciding with the appearance of a muscle artifact on EEG. From eye opening to movement, the patient’s gaze remains fixed with an absence of eye movements on electrooculogram. Lower figure shows video snapshots of behavior. The patient has an arousal followed by eye opening then remains still and staring at a point in the corner of the room. She then briefly lifts her head, touches her face then settles back down to return to sleep.

**Figure 3:**
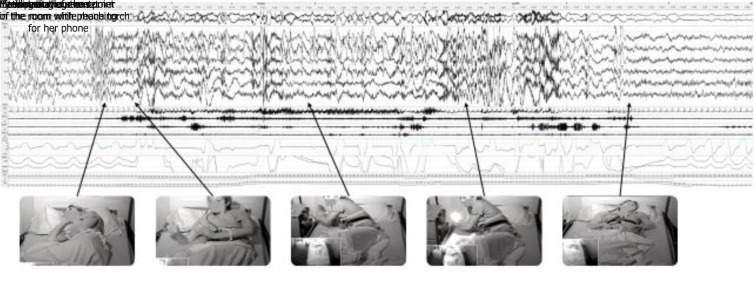
Hallucination episode arising from N3 sleep. Upper figure shows three consecutive 45-second epochs of video-polysomnography (VPSG). Lower figure shows a sequence of snapshots of the event. Eye opening follows arousal by 3 seconds and corresponds with a change in electroencephalogram (EEG) demonstrating mixed frequencies and increase in muscle tone. After a 4-second delay, the patient abruptly raises her head and trunk and begins to stare at a specific point in the corner of the room. She then reaches for her mobile phone to illuminate the corner of the room that she is staring at during the entire episode. Shortly after illumination, the patient returns to a supine position and relaxes (resolution of artifact), and the EEG demonstrates clear alpha rhythm.

Videopolysomnography analysis was only remarkable for the DOA diagnosis, with no other overt sleep pathology identified despite other additional sleep complaints at presentation. In 14 patients (53.5%), classical DOA patterns were recorded during VPSG and were characterized by sudden arousals from slow-wave sleep (Figure 1). However, in 16 (53.5%) patients, alongside classical DOA features, an additional, novel pattern of EEG and behavioral manifestations corresponding to the hallucinatory episodes was identified (Figures 2 and 3).

During DOA episodes, previously described post-arousal EEG patterns (24,25) were identified on the basis of predominant slow or fast activity or intermixed activity between the two. More specifically, we found three distinct patterns: 1) diffuse, rhythmic delta activity; 2) diffuse delta and theta activity intermixed with alpha and beta activity; and 3) prominent alpha and beta activity.

Behavioral patterns were also identified with differing complexities, ranging from simple movements, including eye opening and head raising, to more complex motor behaviors involving ambulation. These patterns are consistent with previous descriptions by Loddo et al (26). Among the group that did not demonstrate the novel pattern, 12 patients had typical DOA behavioral manifestations characterized by simple behaviors (eg, eye opening and head raising). However, complex behaviors including sitting up, interacting with the environment, or attempting ambulation were also noted in 2 patients. Eleven patients experienced more than one episode during the VPSG.

A completely distinct EEG and behavioral pattern was found in 16 patients. This EEG and behavioral pattern differed from the abrupt-onset pattern typically seen in DOA, and it differed from the three typical post-arousal EEG patterns. Specifically, in this group of DOA patients, after an arousal from NREM sleep, patients were seen to open their eyes, stare or visually track while remaining still for a period of time. Subsequent to this, they exhibited motor behaviors of varying complexity, from simple to complex. Out of 16 patients in this group, 12 manifested simple behaviors that involved eye opening and raising the head and or trunk from the pillow. The patients were seen to be staring at a point in the room or visually tracking (Figure 2). Four out of 16 DOA patients demonstrated complex behaviors, which included sitting upright in the bed, appearing to reach out for something, or interacting with the environment (Figure 3). In regards to the post-arousal EEG pattern, in all 16 patients, an EEG arousal was followed by a one- to six-second delay until eye opening. This quiescent behavioral period coincided with diffuse low-voltage mixed-frequency activity comprising theta intermixed with alpha, which remained stable and artifact free for another one to 12 seconds, until the onset of motor behavior obscured the EEG. Concomitantly, a rapid increase in heart rate was observed. Subsequently, once movement and muscle artifact resolved, an awake EEG was observed for up to three minutes, with EEG returning to N1 sleep in all recorded episodes. From initial arousal to the return to sleep, events lasted from between eight seconds to three minutes (Figures 2-3).

VPSGs of 30 DOA patients were further compared based on the distinct EEG and behavioral patterns observed. Specifically, those who demonstrated the novel EEG and behavioral pattern were compared with those who did not.

Differences between the two groups were not significant regarding sleep parameters (Table 2). However, patients who exhibited the novel EEG and behavioral pattern were predominantly female (N = 13). Notably, 7 patients had full recollection of the episodes the morning after the VPSG and they were patients who demonstrated the novel hallucinatory pattern.

**Table 2:** Clinical and video polysomnography findings*

Clinical data	With hallucinations (N = 16)		Without hallucinations (N = 14)		
	N	%	N	%	P
Age in years, mean±SD	33±5.18		35±7.35		0.667
Gender (M-F)	13-3		8-6		0.035
Recall	7	43.75	0	0	
>1 episodes	12	75	11	78.6	
DOA pattern					
simple behavior	13	81.2	12	85.7	
complex behavior	3	18.8	2	14.3	
Hallucination pattern					
simple behavior	12	75	n/a	n/a	
complex behavior	4	25	n/a	n/a	
Sleep parameters, median (IQR)					
Total recording time, min	484.6 (471.1-531.9)		472.5 (449-506.7)		0.131
Sleep period, min	460.8 (449.1-507.3)		452.8 (422.5-493.8)		0.58
Total sleep time, min	407.2 (377.1-460.8)		392 (358.9-429.1)		0.473
Wake after sleep onset, min	54.6 (37.3-73)		59.3 (33.8 -109.6)		0.822
Sleep latency, min	21.8 (13.5-28.4)		12.8 (8.9-20.3)		0.142
Sleep efficiency, %	85.1 (813-88.3)		79 (72.6-89.3)		0.4
Stage N1 sleep, %	8.3 (5.7-11.2)		12.3 (4.1-13.7)		0.44
Stage N2 sleep, %	41.3 (34.8-48.6)		44.2 (38.1-54)		0.423
Stage N3 sleep, %	27.8 (22.3-33.2)		24.8 (18-27.2)		0.8
Rapid eye movement sleep, %	20.4 (18.2-24.5)		21 (17.7-23.3)		1
Arousal index, per hour	15.7 (11.5-21.2)		13.5 (8.1-16.2)		0.2
Apnea hypopnea index, per hour	0.7 (0.3-1.9)		0.4 (0.0-3.1)		0.88
Oxygen desaturation index, per hour	0.4 (0.0-1.1)		0.5 (0.0-2.4)		0.64
Periodic limb movement index, per hour	1.6 (0.2-6.9)		0.7 (0.0-6)		0.38

*Abbreviations: DOA – disorder of arousal; SD – standard deviation; IQR – interquartile range.

Some examples of complex hallucinatory experiences include the following episodes. For instance, during one event, the patient grabbed their t-shirt that was near their pillow and repeatedly swatted at something in the air. During this episode they were heard to shout and swear and appeared quite distressed. In the morning, they recalled the episode and described seeing a sinister person in the room by the wardrobe that began to move toward them. Another patient was seen to stare at a specific spot in the corner of the room, in the dark, before raising their head and trunk while continuing to stare at the same point. They then reached for their mobile phone to turn on the torch and shone the light in the direction of their gaze (Figure 3). Shortly after illumination, the patient laid down in the supine position and relaxed. The EEG correlate subsequently showed awake alpha rhythm. In the morning they recalled having seen children in the room.

DOA events in all 30 patients were recorded during the first two NREM cycles in VPSG but there was no identifiable temporal relationship between the events (between typical DOA and the hallucinatory episodes). All sleep parameters and arousals characteristics are shown in Table 2.

## Discussion

This study showed for the first time a distinct post-arousal EEG and behavioral pattern in patients with DOA during their ongoing hallucinatory experiences. This pattern significantly differs from the traditional VPSG pattern of parasomnic events. Thus, it can be hypothesized, that as such, it may reflect the underlying mechanism of ongoing hallucinatory experiences in the DOA patients.

In this study, in all 16 patients, the EEG and temporal semiology of recorded hallucinations was strikingly similar. All events were from N3 sleep, and shared a similar post-arousal EEG and temporal behavioral pattern. For instance, all patients, after a varying delay, appeared to open their eyes and to visually track for a period of time before more complex behaviors started. The distinct post-arousal EEG pattern comprised of diffuse, initially mixed, low-voltage frequencies including theta and alpha, which subsequently progressed into wake EEG activity. This is in sharp contrast with the typical abrupt-onset and the post-arousal EEG patterns that are historically described and associated with DOA episodes (24,25). These results may support the assertion that in patients suffering from DOA, hallucinatory experiences may represent other features of a dissociation of states occurring during the DOA episodes.

DOA represent a form of sleep-wake state dissociation (2,11,27,28). During transitions between NREM sleep and wakefulness, in predisposed subjects, a dissociation of states can occur among different brain structures manifesting as DOA episodes (2,11). It has been similarly argued that DOA may be disorders of transition from slow-wave sleep to REM sleep (29,30). The common agreement would state that dissociation between states can be the key feature of several sleep disorders (3,5,7). The dissociation may rise from prevailing wakefulness, prevailing NREM sleep, and from REM sleep, and it is caused by the intrusion of features of other stages into the ongoing state (6,31). For instance, sleep-related hypnagogic and hypnopompic hallucinations are considered to be dissociative phenomena as well, in which REM imagery intrudes into wakefulness or into a relatively high level of arousal (32), most likely at the transition between wakefulness and sleep and *vice versa* (6). They represent one of the cardinal features of narcolepsy (17,33), although they also occur in around 25%-37% of the general population (34), and are difficult to differentiate from dream-related experiences (15).

Another distinct form of sleep-related hallucinations are the so-called CNVH (1), characteristics of which are shared by this group of patients. However, CNVH are commonly associated with other central nervous system pathologies, including midbrain and diencephalic disorders (peduncular hallucinosis) (35-37), visual impairment (eg, Charles Bonnet syndrome) (38), and neurological disorders (Parkinson’s disease and Lewy body dementia) (37,39), which were not present in this group of patients. Nonetheless, some patients with CNVH also have comorbid NREM parasomnias (40). CNVH often take the form of vivid mobile or immobile images of animals or people, sometimes distorted in shape, typically occurring following a sudden awakening and often, they are initially perceived as being real and frightening (1,21,41). CNVH have also been reported to cease with an increase in the background illumination (1). In keeping, 80% of this cohort of patients reported disappearance of imagery when turning lights on. This may suggest that illumination levels, visual processing mechanisms, as well as arousal mechanisms and the length of the events moderate hallucinatory experiences and their subsequent recall (19,37,38). It is therefore possible that the episode resolves with the increased level of arousal, with gained insight, and the recovery of the visual input.

Furthermore, among the patients reporting to “suddenly see” things at varying frequencies during the night, only 7 of the 16 patients reported having had their usual episodes during the VPSG. The 4 patients who exhibited complex motor behavior during the episodes reported full recall of the visual hallucination the morning after the VPSG. It may be hypothesized that the complexity of the behaviors and the varying duration of the succeeding period of wakefulness may explain the subjective differences in the recall or the amnesia for it.

Of note, 23.3% of patients recalled having structured, narrative dreams, which were related to post-arousal hallucinatory experiences (42). Dream mentation may be present in DOA, and some studies have supported the hypothesis that DOA may represent a form of dream-enacting behavior (12,43). More interestingly, patients in this study reported that dream imagery that preceded the hallucination episodes was related to the visual hallucinatory experiences. The dream imagery was then perceived to be real, superimposed, and to exist in the real world environment. While this is beyond the scope of this study, future studies should elucidate if post-arousal hallucinatory experiences reflect a juxtaposition of dream and real world imagery.

It can be posited that in predisposed subjects, in delicate conditions of transition of states, an admixture of wake, NREM, and possibly also REM sleep elements can coexist until a complete, full wakefulness is reached, manifesting as hallucinatory experiences (42). However, in contrast to the abrupt onset typically seen in the DOA events, during the hallucinatory phenomena, the progression of events appears to be more gradual, and more transition errors of switching between different states may take place, arguably allowing for a more prolonged flux between different brain sleep states.

The coexistence of different states may result in the images of the sleeping mind becoming incorporated and projected into the real-world environment, so that they are visualized upon the surroundings until full wakefulness and a full insight into their hallucinatory nature are achieved.

In conclusion, this study advances and describes an important and a distinct post-arousal EEG and behavioral pattern, which was found in patients with DOA who concomitantly experienced ongoing hallucinations. This pattern differs from previously described post-arousal patterns, and likely reflects the acute nature of recorded hallucinations during DOA events and may be a useful, additional diagnostic tool in clinical practice.

However, there are several noteworthy limitations to this small, retrospective study of DOA events, which detract from finite mechanistic inferences. For instance, one-night vPSG may misrepresent the frequency and the nature of sleep pathology. Thus, it is possible that this particular pattern is either more or less prevalent than the study findings suggest. Furthermore, medications and other sleep, neurological, and psychiatric disorders might also influence the occurrence of parasomnias. Notably, in this study only one patient was taking treatment that could potentially modify the sleep structure and the occurrence of sleep disorders. Morning questionnaires may pose another important limitation. It is uncertain how much of patient recall may be lost and or confabulated unless collected immediately post DOA event, and future prospective studies are required to investigate this intriguing nocturnal phenomenon further. It is hoped that by learning more about any involved neurocircuitry, it may be possible to shed light onto the pathophysiological mechanisms of the hallucinations experienced in other disorders.
