# Supplementary material for: Nocturnal visual hallucinations in patients with disorders of arousal: a novel behavioral and EEG pattern
Source: Croat Med J. 2022 Oct;63(5):438–47. doi: 10.3325/cmj.2022.63.438 (PMC9648088; doi:10.3325/cmj.2022.63.438)
Supplement: Supplementary Table 1 [file CroatMedJ_63_s004.pdf]

Supplementary Table 1.

Example Questions post sleep study questionnaire

Name: Date of Birth:

<table>
<tr><td colspan="4">In brief, what is your main sleep complaint?</td></tr>
<tr><td>Did you experience this during your sleep study?</td><td>Yes</td><td>No</td><td>Don’t remember</td></tr>
<tr><td>How did you sleep compared to a normal night at home?</td><td>Worse than normal</td><td>About the same</td><td>Better than normal</td></tr>
<tr><td>How long do you think it took to fall asleep?</td><td colspan="3"></td></tr>
<tr><td>Did you experience a typical event during the sleep study?</td><td>Yes</td><td>No</td><td>Don’t remember</td></tr>
<tr><td>Do you think it was a dream?</td><td>Yes</td><td>No</td><td>Don’t remember</td></tr>
<tr><td>Did you see anything (e.g. animals, insects, people, shadows)?</td><td>Yes</td><td>No</td><td>Don’t remember</td></tr>
<tr><td>Did they change in shape, move or did you feel threatened by them?</td><td>Yes</td><td>No</td><td>Don’t remember</td></tr>
<tr><td>Did you feel, smell or taste anything?</td><td>Yes</td><td>No</td><td>Don’t remember</td></tr>
<tr><td colspan="4">If you had an event, do you remember how you were feeling (e.g. anxious, frightened, hot)?</td></tr>
<tr><td colspan="4">Do you have any further comments regarding your study?</td></tr>
</table>
